# Circulation of SARS-CoV-2 and Co-Infection with *Plasmodium falciparum* in Equatorial Guinea

**DOI:** 10.3390/idr17050111

**Published:** 2025-09-10

**Authors:** Diana López-Farfán, Policarpo Ncogo, Consuelo Oki, Matilde Riloha, Valero Ondo, Pablo Cano-Jiménez, Francisco José Martínez-Martínez, Irene Molina-de la Fuente, Iñaki Comas, Nerea Irigoyen, Pedro Berzosa, Agustín Benito Llanes, Elena Gómez-Díaz

**Affiliations:** 1 Instituto de Parasitología y Biomedicina López-Neyra, Consejo Superior de Investigaciones Científicas (IPBLN, CSIC), 18016 Granada, Spain; elena.gomez@ipb.csic.es; 2 Instituto de Salud Carlos III, 28029 Madrid, Spain; 3 Fundación CSAI, 28029 Madrid, Spain; pberzosa@isciii.es; 4 Ministerio de Sanidad y Bienestar Social, QQ2H+8WC Malabo, Equatorial Guinea; 5 Tuberculosis Genomics Unit, Instituto de Biomedicina de Valencia, Consejo Superior de Investigaciones Científicas (IBV, CSIC), 46010 Valencia, Spain; pcano@ibv.csic.es (P.C.-J.); fmartinez@ibv.csic.es (F.J.M.-M.);; 6 Centro Nacional de Medicina Tropical, CIBERINFEC, Instituto de Salud Carlos III, 28029 Madrid, Spain; 7 CIBER de Epidemiología y Salud Pública, CIBERESP, 28029 Madrid, Spain; 8 Division of Virology, Department of Pathology, University of Cambridge, Cambridge CB2 1QP, UK

**Keywords:** COVID-19, malaria, co-infection, coronavirus, Africa, genomic surveillance

## Abstract

**Background/Objectives:** The impact of COVID-19 in Africa has been controversial. Data from African countries are heterogeneous and generally scarce. Many regions are also highly endemic for other infectious diseases like malaria, and it has been suggested that the low incidence and mortality of COVID-19 in malaria-endemic regions could have been related to cross-immunity between malaria and SARS-CoV-2. The aim of this study was to determine the prevalence of SARS-CoV-2 and circulating variants as well as the frequency of co-infections with malaria in Equatorial Guinea. **Methods:** We conducted antigen tests for SARS-CoV-2 and microscopy malaria examinations in 1556 volunteer participants at six health centres in Bioko and Bata from June to October 2021 and performed SARS-CoV-2 whole-genome sequencing on positive samples to determine the diversity and origin of circulating variants. **Results:** We report 3.03% of SARS-CoV-2 and 22.25% of malaria prevalence over the sampling period; SARS-CoV-2 cases were found at a similar frequency in all age groups, whereas malaria was most frequent in children and teenagers. Six cases of malaria and SARS-CoV-2 co-infection were found, representing 0.37% prevalence. Genome sequences of 43 SARS-CoV-2 isolates are reported, most of which belong to the lineage Delta and, according to pandemic-scale phylogenies, were introduced from Europe on multiple occasions. **Conclusions:** This study is relevant in providing first-time estimates of the real prevalence of SARS-CoV-2 in this malaria-endemic country, with the identification of circulating variants, their origin, and co-occurrence with malaria. These data regarding the impact of the pandemic and co-infection with endemic diseases are relevant in future pandemics preparedness.

## 1. Introduction

The coronavirus disease 2019 (COVID-19) caused more than 772 million cases and nearly 7 million deaths worldwide. However, only a small proportion of these (around 9.5 million cases [1.24%] and 175,473 deaths [2.51%]) were reported in Africa (data as of December 2023, World Health Organization (WHO) COVID-19 Dashboard) [1]. Moreover, inside Africa, the distribution of cases has been uneven. Most of the reported cases were from a few Northern and Southern countries like South Africa (4 million cases), Morocco (1.27 million cases), and Tunisia (1.15 million cases). In comparison, only 634,161 cases accounted for eleven Central African countries. These data suggest country-specific factors such as reduced transmission or limited testing, raising questions about the real impact of the pandemic in Central African countries.

Genomic SARS-CoV-2 surveillance has been vital in monitoring the emergence, evolution and spread of different variants of concern (VOCs). Several VOCs, such as Alpha, Beta, Delta and Omicron, were first detected on the African continent or spread extensively on it [2,3,4]. Despite the heterogeneous pandemic across Africa, most countries have reported multiple waves of infection [4]. Genomic sequencing has revealed that each wave has been dominated by a different lineage [4,5]. Within Africa, regional viral population diversity has been observed in the first and second waves and, to a lesser extent, in the third wave. In North and Southern Africa, the first and second waves were dominated by Beta (B1) and Alpha lineages, replaced by Delta and Omicron in the third and fourth waves, respectively. However, different lineages in West, East, and Central Africa have dominated the first, second, and third waves [4]. In addition, other minor lineages were detected co-circulating and contributing to epidemic waves in all regions of the continent [4,6]. In this context, characterizing the local diversity of SARS-CoV-2 variants on a country-by-country basis becomes necessary. Furthermore, there is an ever-present and increasing risk of emerging and re-emerging infectious diseases due to trends such as urbanization and climate change. In this context, precise data of the impact of COVID-19 and data of co-infection with other endemic diseases are important for future pandemics preparedness.

Equatorial Guinea is located on the west coast of Central Africa and consists of two parts, the mainland and an insular region. As of December 2023, this country had reported 17,130 cases and 183 deaths, most of them from the insular region (77.2%) [7]. Like in other African countries, data about the prevalence of SARS-CoV-2 in Equatorial Guinea have been scarce. Indeed, to our knowledge, no representative studies exist that have evaluated the prevalence of SARS-CoV-2 in this country.

Since Equatorial Guinea confirmed its first case of COVID-19 community transmission on 14 March 2020, there have been five waves [7]. A genomic analysis of SARS-CoV-2 revealed that the first wave from April to July 2020 was dominated by the early lineage B.1.192, the second wave from January to April 2021 was dominated by the Beta variant (B.1.351), and the third wave began during July 2021 with the Delta variant (AY.43) [5].

Before this study report, only 213 SARS-CoV-2 whole-genome sequences from Equatorial Guinea were deposited in the GISAID database [8]. These data correspond, however, to 1.2% of reported cases, which is far from the WHO recommendation to sequence at least 5% of COVID-19 cases by country [9]. Hence, there is a need to increase the number of SARS-CoV-2 genomes to sequence for a complete assessment of the diversity of variants that have been circulating in the country.

Apart from the direct mortality impact of COVID-19, the pandemic has also resulted in a worsening crisis in controlling several endemic diseases in Africa [10,11,12]. During the first year of the pandemic, malaria cases and deaths in Africa increased from 213 to 228 million cases and from 534,000 to 602,000 deaths (15 million more cases and 68,000 more deaths). This was mainly due to disruption to malaria interventions [13], reaching the worst-case scenario projected at the beginning of the pandemic [14]. During 2021, the increase in malaria cases continued but was less marked (two million more cases), while deaths remained stable [13]. This was possible due to the efforts of countries and partners that provided additional funding to deliver essential malaria services [13].

Besides the collateral effects on endemic disease control programmes, the circulation of SARS-CoV-2 in malaria-endemic countries raises the possibility of co-infection. The frequency and health consequences of co-infection at the population level have been assessed in eight African malaria-endemic countries [15,16,17,18,19,20,21,22,23,24,25,26] and one Indian [27] one. However, these studies have shown very heterogeneous results depending on the study design and the population or subpopulation sampled (Supplementary Table S1).

Equatorial Guinea is among the moderate-to-high-malaria-endemic countries in the African Region, accounting for 95% of cases and 96% of malaria deaths globally. Malaria transmission in Equatorial Guinea is seasonal, with peaks from June to October. To date, no study has examined the occurrence and frequency of COVID-19 and malaria co-infection.

To fill these gaps, in this work, we conducted a cross-sectional study of 1556 participants at six health centres, three on Bioko (an island) and three in Bata (the mainland), from June to October 2021, coinciding with the seasonal peak of malaria transmission. All participants were tested for SARS-CoV-2 by rapid antigen diagnostic tests and for malaria by microscopy. Moreover, swab samples were taken for SARS-CoV-2 RT-qPCR and whole-genome viral sequencing. To our knowledge, this is one of the largest SARS-CoV-2 epidemiological studies conducted in Sub-Saharan Africa and is also the first to evaluate SARS-CoV-2 and malaria co-infection in this malaria-endemic country.

## 2. Materials and Methods

### 2.1. Study Design

This observational cross-sectional study was conducted at six health centres in Equatorial Guinea, three located on Bioko Island (Hospital Regional de Malabo, Centro de Salud Buena Esperanza and Centro de Salud de Campo Yaundé) and three located in the mainland city of Bata (Centro de Salud María Rafols, Centro de Salud La Libertad and Hospital Regional de Bata). The study participants were patients attending the different health centres independently of their symptomatology (samples were collected from people with and without symptoms) who were approached by the health workers collaborating with the study and invited to participate. Participation in the study was voluntary. A total of 1556 participants were enrolled from 1 June to 4 October 2021 (Supplementary Table S2). Inclusion criteria included (1) not having been vaccinated against SARS-CoV-2 and (2) being at least 5 years old. The demographic and clinical information collected included age, gender, temperature, symptomatology, and pregnancy state. Prior to the taking of any samples, participants were required to sign the informed consent form. For each person, one drop of blood was obtained by capillary puncture of the fingertip and was used for *P. falciparum* detection by microscopy. A nasopharyngeal sample was also collected for antigen detection for SARS-CoV-2. For SARS-CoV-2-positive antigen test participants, a second nasopharyngeal sample was taken, and the swab was stored in pre-filled tubes containing 0.5 mL of inactivating lysis buffer (AVL, QIAGEN, Venlo, The Netherlands) for the molecular testing of SARS-CoV-2 and whole-genome viral sequencing. These samples were kept at 4 °C for 24 h and at −20 °C or −80 °C for extended storage and subsequent analysis.

### 2.2. Malaria and SARS-CoV-2 Diagnosis

Malaria detection was performed by microscopy examination, considered as the gold standard for large-scale diagnosis. Blood thick smears were screened for *Plasmodium falciparum* asexual and gametocyte stages by experienced technicians. Previous studies in Equatorial Guinea reported a sensitivity for this method of 50–500 parasites/μL [28]. Antigen detection of SARS-CoV-2 was performed from individual nasal swab samples using the Panbio™ COVID-19 Ag Rapid Test (Abbot, Chicago, IL, USA) following the manufacturer’s instructions. For SARS-CoV-2 molecular detection, RNA was extracted from 140 µL of the AVL preservative solution using the QIAamp viral RNA extraction kit (QIAGEN) following the manufacturer’s instructions. RT-qPCR analysis was performed using the one-step Direct SARS-CoV-2 Realtime PCR Kit (Vircell S.L, Granada, Spain) following the manufacturer’s instructions; reactions were performed in 20 µL final volume (5 µL of RNA sample and 15 µL of RT-PCR mix) using a CFX96 Real-Time PCR Detection System (Bio-Rad, Hercules, CA, USA). Cycle threshold (Ct) values were analyzed using the Bio-Rad CFX manager software 3.1. Gene targets identified with were the nucleocapsid (N), the envelope (E), and the human RNase P. Samples were considered positive when the N and E target genes had a cycle threshold (Ct) < 40. Positive and negative controls were always included.

### 2.3. Data Analysis

All data was managed in Excel before being exported to R v4.0.0 for analysis. Prevalence of each infection and co-infection was calculated by dividing confirmed cases by population screened according to age and gender, then it was adjusted according to their weight in the population considering national data from 2021 [29] (Supplementary Table S3). The association between variables was tested with the Wilcoxon rank sum tests (for continuous quantitative variables) and the Kruskal–Wallis chi-squared test or Fisher exact test as appropriate. A statistically significant multivariable logistic regression model (*p*-value < 0.01), compared with the null model by ANOVA test, was built for each infection independently and for co-infection. The model combines one continuous variable (age) and four categorical variables. All analysis used a 95% confidence level and a *p*-value of <0.05 for statistical significance.

### 2.4. Whole-Genome Sequencing and Phylogenetic Analysis

RNA from SARS-CoV-2-positive samples with a Ct value ≤ 35 was sent for sequencing to the Instituto de Biomedicina de Valencia (IBV-CSIC). RNA was retrotranscribed into cDNA, and SARS-CoV-2 complete genome amplification was performed in two multiplex PCR, accordingly to an openly available protocol developed by the ARTIC network5 using the V4.1 multiplex primers scheme (artic-network n.d.). Two resulting amplicon pools were combined and used for library preparation. Genomic libraries were constructed with the Nextera DNA Flex Sample Preparation kit (Illumina Inc., San Diego, CA, USA) according to the manufacturer’s protocol with five cycles for indexing PCR. Whole-genome sequencing was performed in a MiSeq platform (2 × 150 cycles paired-end run; Illumina, San Diego, CA, USA). Sequences obtained went through an open-source bioinformatic pipeline based on IVAR6 [30]. The steps of the pipeline are as follows: (1) removal of human reads with Kraken; (2) filtering of the fastq files using fastp v 0.20.1 (arguments: –cut tail, –cut-window-size, –cut-mean-quality, -max_len1, -max_len2); (3) mapping and variant calling using IVAR v 1.2; and (4) quality control assessment with MultiQC [31]. Consensus sequences generated by this pipeline were aligned against the SARS-CoV-2 reference sequence [32] with MAFFT [33]. Problematic positions were masked using the mask_alignment.py script from the repository maintained by Rob Lanfear7. Lineages and clade nomenclature were assigned using Phylogenetic Assignment of Named Global Outbreak Lineages, PANGOLIN [34], and Nexclade online tool [35]. The phylogenetic tree was generated with Nextclade v2.5.08, downloaded in JSON format, and visualized using Auspice v2.37.3 online tool9 [36,37].

### 2.5. Genome-Based Epidemiological Analysis

A genome-based epidemiological analysis was performed on the sequenced Guinea Equatorial samples following the pipeline described in [38]. We used a pandemic-scale phylogenomic analysis tool called UShER [39] to place the newly sequenced samples in a prebuilt phylogeny. For this purpose, we downloaded an existing SARS-CoV-2 phylogeny (eTree) composed of 15,649,343 sequences uploaded in GISAID [8] on 27 July 2023 (available upon request). This method avoids subsampling the dataset by the country of origin or the sampling date, maintaining the broadest possible context. Using Robert Lanfear’s global_profile_alignment.sh [40], we aligned the Guinea Equatorial sequences against the Wuhan reference sequence (MN908947.3). We used the faToVcf tool from the UShER toolbox to obtain the VCF file and used this file as input along with the eTree to place the new 43 sequences.

To speed up the analysis, we extracted the Beta and Delta variants’ clades from the eTree using matUtils [41] after identifying the parental nodes using the Taxonium viewer [42]. Finally, using a custom code developed in R, we identified the likely country/region of origin and the period of the introductions. To do so, we first defined 3 possible categories for our sequences: transmission group, if they fell in a node in which >60% samples were from Equatorial Guinea; unique sequence, if they fell in a node with lower presence of the country and the length of their branches was greater than 0; and unclassified, if they had no branch length. Then, we searched for the closest sequence with the minimal genetic distance in SNPs that was collected within the 3-month period prior to the sampling date and the sampling date minus 10 days. All the commands used in this analysis are available at the GitLab repository, along with the custom code (https://gitlab.com/tbgenomicsunit/covid-guinea) (accessed on 16 August 2023).

## 3. Results and Discussion

Compared to the rest of the world, the impact of COVID-19 in Africa has been relatively low in terms of severe disease incidence and mortality rate. Specific socio-ecological and economic factors (i.e., warm weather, younger population, lower population density and mobility, limited testing capacity, and previous training of immunity due to past infectious diseases) have been proposed to explain the apparent lower transmission [43,44,45]. Other reports, however, suggest that this impact has been vastly underestimated, especially in low-income countries, due to insufficient testing and uneven genomic surveillance. This is probably the case in Equatorial Guinea, where no comprehensive studies have assessed the prevalence of SARS-CoV-2 and where the sequencing availability has been very low.

Apart from the noticeable direct effects, the COVID-19 pandemic severely affected the global fight against several endemic infectious diseases, namely, VIH, tuberculosis, and malaria, mainly because of an interruption and reduction in their control programmes [10,11,12]. But recent data points towards a more direct biological interaction between these diseases. In the case of malaria, it has been suggested that severity of COVID-19 infection in malaria-endemic regions may be influenced by co-infection or previous malaria exposure [22,27]. Some studies also suggested a possible cross-immunity between the malaria parasite and SARS-CoV-2 [46,47,48,49]. Prevalence studies of SARS-CoV-2 and co-infection with malaria in sub-Saharan countries are therefore essential to understand the real impact of the pandemic on the African continent.

This is the first comprehensive study of SARS-CoV-2 prevalence performed in Equatorial Guinea and the first to evaluate malaria co-infection. For this purpose, 1556 participants were recruited at six Equatorial Guinea health centres during four months in 2021 (from June to October) (Supplementary Table S2). The study was conducted in three health centres on Bioko Island (the site of the country capital, Malabo) and three health centres in the mainland city of Bata. Of the participants, 72.6% were female and 27.4% were male. Samples were grouped into four age groups, as follows: A. 5–12 years old (16.8%), B. 13–20 years old (20.8%), C. 21–40 years old (46.2%), and D. >40 years (16.3%). The distribution of positive cases by age and gender is detailed in Table 1. To correct for any sampling bias in our dataset, the prevalence of infections was adjusted by age and gender considering publicly available Equatorial Guinea population national data from 2021 [29] (Supplementary Table S3).

**Figure 1:**
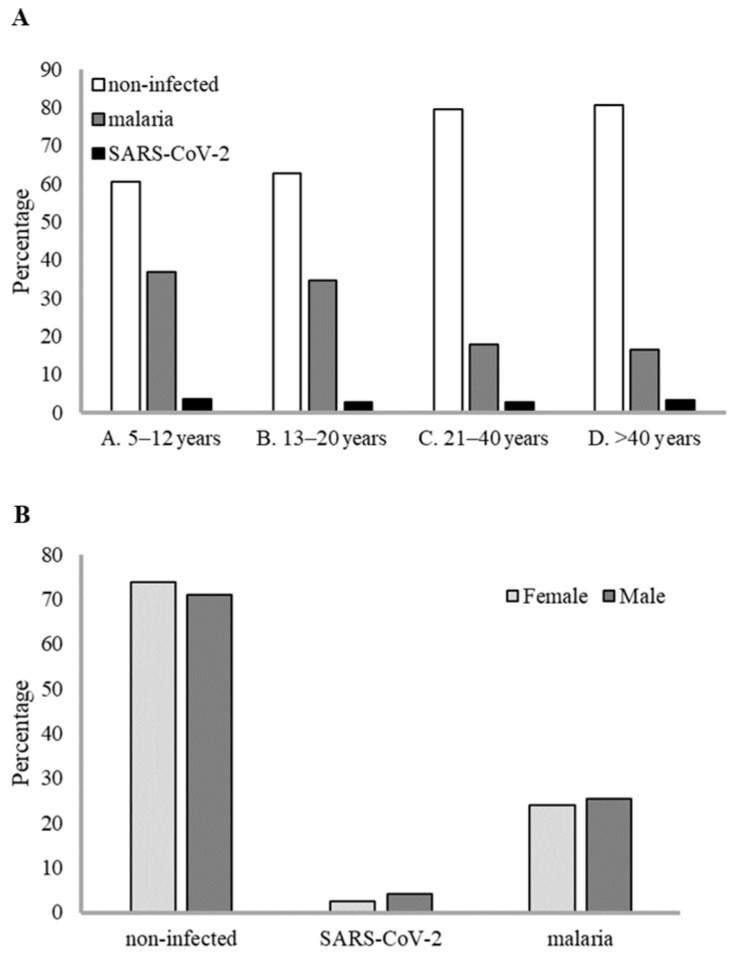
Prevalence of SARS-CoV-2 and malaria cases by age group and gender. (**A**) Prevalence by age group. (**B**) Prevalence by gender.

**Figure 2:**
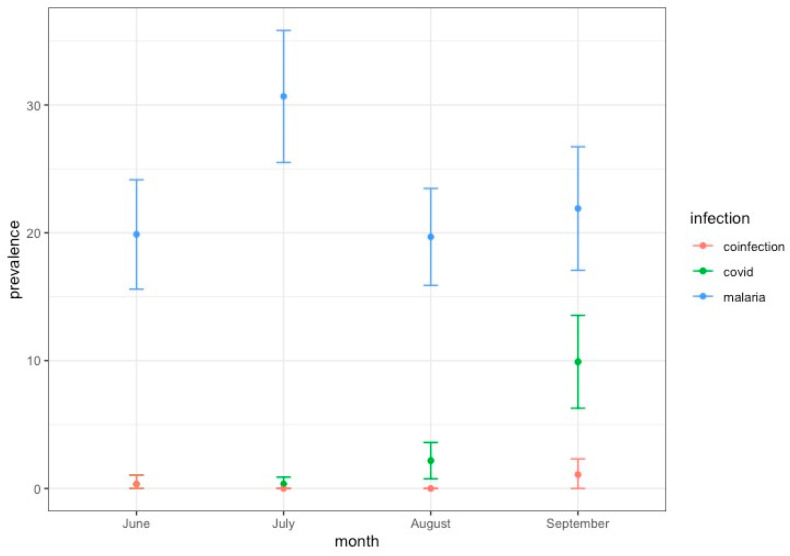
Prevalence of SARS-CoV-2 and malaria over the study period.

For this sampling period and subjects, a total of 46 individuals tested positive by SARS-CoV-2 antigen test, resulting in an overall adjusted prevalence of 3.03% (95% CI 2.1–3.9). However, most of the positives were at the health centres of Bioko Island, resulting in a local prevalence of 6.7% (43 positives out of 643 samples) (Supplementary Table S2). This agrees with the official data from Equatorial Guinea authorities reporting that most SARS-CoV-2 cases were from Bioko Island [7], the site of the capital. SARS-CoV-2-positive cases were found at a similar prevalence in all age groups (Figure 1A), and there were no significant differences between males and females (W = 39812, *p*-value = 0.07) (Figure 1B). A temporal trend for SARS-CoV-2 positives increased progressively over the sampling period to reach a peak of 10% of prevalence in September 2021 (Figure 2). This trend agrees with the official reports for this country that recorded a peak in cases by September 2021 [50]. Regarding the symptomatology, most of the SARS-CoV-2-positive cases were mildly symptomatic (91.3%), with fever as the most common symptom (Supplementary Tables S4 and S5). Indeed, our multivariable binomial regression model analysis indicates a significant association of SARS-CoV-2 infection only with fever (*p* = 0.021) and Bioko origin (*p* < 0.001) (Supplementary Table S6).

**Figure 3:**
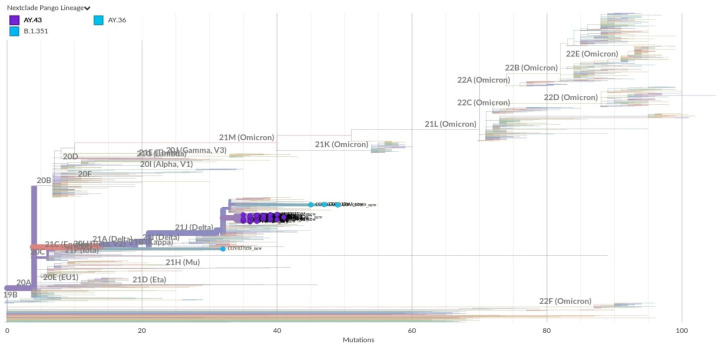
Phylogenetic tree with the 43 new SARS-CoV-2 genomes from Equatorial Guinea. Equatorial Guinea genomes placed on a reference tree of 1495 published genomes from all over the world. The new sequences are represented as dots; blue (lineages AY.36; B.1.351) and purple (lineage AY.43). Phylogenetic tree generated with Nextclade online software v2.5.0 (https://clades.nextstrain.org) (accessed on 1 June 2023) and visualized using Auspice v2.37.3 (https://auspice.us/) (accessed on 1 June 2023).

A total of 43 SARS-CoV-2 positive nasopharyngeal swabs were confirmed by RT-qPCR with high viral loads (Ct values in the range of 7–29). The RNA of these 43 PCR-positive samples was sent for whole-genome sequencing. After genome assembly and quality control, good-quality genomes were obtained from 40 samples, and medium/low-quality genomes were obtained from three samples. All the sequences were assigned to lineages using the dynamic lineage classification method PANGOLIN [34]. Phylogenetic analysis revealed that most of the sequences belonged to the lineage AY.43 (38 samples); however, other minor lineages were found, like AY.36 (3 samples) and B.1.351 (2 samples) (Figure 3 and Supplementary Table S7). Our results are in line with a recent genomic study that described the SARS-CoV-2 lineages for the first three waves of the pandemic in Equatorial Guinea from February 2020 to October 2021 [5].

Our sampling period began in June 2021, when a few positive cases were detected, and coincided with the end of the second wave (Figure 2). From the end of June to the first half of July, the two genomes available were identified as Beta VOC (B.1.351). This lineage was first identified in South Africa in September 2020 and subsequently defined as a variant of concern (VOC) [3]. Since then, it spread to other African countries [51]. The earliest sequence of this lineage reported from Equatorial Guinea was in January 2021 [52]. However, the peak of our sampling mostly overlaps with the third wave of the pandemic, between August and September 2021 (Figure 2). For that period, most isolated sequences were identified as the Delta variant (AY.43), the VOC that dominated the third wave in Equatorial Guinea. The Delta variant was first detected in India in late 2020 [53], from where it was spread to more than 170 countries, including 37 African territories [4]. It has been reported that most of the introductions of this variant into Africa (72%) can be attributed to India [4]. The other minor lineage found in this study was AY.36, corresponding to samples collected at the end of August. This is an uncommon Delta lineage that was dominant in Nigeria by June-August 2021 (80% cases) but that represented less 0.5% of Delta cases globally as of September 2021 [6].

**Figure 4:**
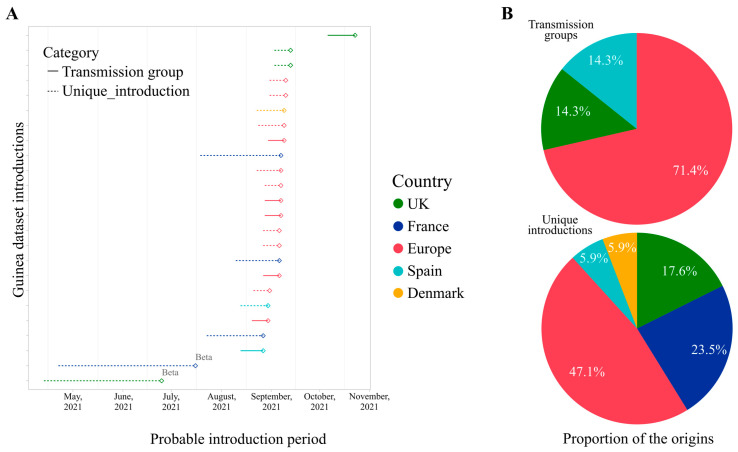
Origin and introduction period of the Equatorial Guinea sequenced SARS-CoV-2 samples. (**A**) Dot plots display introduction periods for transmission groups and unique sequences. Each dot and its segment represent an introduction and its estimated time interval of entrance in the country, coloured by the origin. (**B**) Pie charts represent the proportion of introductions for transmission groups (top pie) and unique introductions (bottom pie).

To better determine the likely origin and introduction period of the Equatorial Guinea sequenced samples, we performed a genome-based epidemiological analysis using pandemic-scale phylogenies [38]. The results are summarized in Figure 4; our script identifies 17 unique introductions and 7 transmission groups or clusters, whereas 11 out of 43 samples remain unclassified. Regarding the origin of the introductions, both the transmission groups and unique introductions are mostly of European origin (5/7, 71.4% and 8/17, 47.1%, respectively); in these cases, we cannot identify the exact country since many potential origins were found.

In the cases where the country of origin was identified, we found one transmission group from the United Kingdom (UK) and another from Spain. Concerning nine unique introductions identified by country, they came from France (4/17, 23.5%), the UK (3/17, 17.6%), Spain (1/17, 5.9%), and Denmark (1/17, 5.9%). The introductions from Spain correspond to the three AY.36 samples, the two Beta (B.1.351) samples came from the UK and France, and the 27 identified AY.43 samples belonged to Europe (16/27), France (7/27), the UK (3/27), and Demark (1/27). Collectively, these results suggest that the introduction of SARS-CoV-2 to Equatorial Guinea occurred from outside Africa. Whether the introduction was from neighbouring countries is much more difficult to evaluate, given the uneven sequencing coverage from Sub-Saharan African countries at the time of analysis. Our results also agree with what has been described in Mozambique during a similar sampling period [38].

Regarding the estimated time interval of the introductions, Beta introductions present a wider interval when compared with Delta, with a range of two to three months of uncertainty (Figure 4). However, this may be due to the large-scale sequencing effort for Delta compared to Beta (~39 K Beta sequences vs. ~5 M Delta sequences in GISAID). In our analysis, most Delta introductions occurred between mid-July and mid-September, in concordance with the Delta wave seen at a global scale.

Regarding malaria prevalence in Equatorial Guinea over the sampling period, 379 individuals were positive for *P. falciparum* by microscopy, resulting in an adjusted prevalence of 22.25% (95% CI 20.09–24.41), and no significant sex difference was observed (W = 226,149, *p*-value = 0.6) (Figure 1B). However, there were significant differences between age groups in malaria prevalence (Kruskal–Wallis chi-squared = 64,812, df = 3, *p*-value < 0.001), with malaria mainly affecting children aged below 12 years (36.8% [95% CI 30.9–42.7]) and teenagers between 13 and 20 years old (34.7% [95% CI 29.5–39.9]) (Figure 1A). The majority of the malaria-positive individuals were symptomatic (92.3%), and the most common symptom was fever (≥37.5), but other mild-to-moderate symptoms were included, such as headache, weakness, muscle and joint pain, cough, dizziness, vomiting, diarrhea, and anorexia (Supplementary Tables S4–S6). No comorbidities were reported among the malaria-positive cases.

We report six cases of confirmed SARS-CoV-2 co-infection with malaria, three in Bata and three in Bioko Island. Of them, two were children (8 and 10 years old), one was a teenager (14 years old), and three were adults (25, 33 and 41 years old) (Table 1). Most of the co-infections were detected during the peak of SARS-CoV-2 cases in September 2021 (Figure 2). The sequenced co-infection cases were the dominant lineage AY.43 (four samples) and B.1.351 (one sample) (Supplementary Table S7). One case was not sequenced because the swab sample could not be obtained. Five co-infection cases were symptomatic; the symptoms registered for three cases were mild and included fever, weakness, headache, and dizziness. For the other two cases, the symptoms were not specified (Supplementary Tables S4–S6).

The overall co-infection-adjusted prevalence reported in this study is low, at 0.37% (6/1556). However, the co-infection prevalence among the malaria-positive subpopulation corresponds to 1.58% (6/379), while the co-infection prevalence among the COVID-19 subpopulation is 13.04% (6/46). This suggest that the prevalence varies depending on the subpopulation studied, being higher in the COVID-19-infected subpopulation.

Apart from this study, SARS-CoV-2 and malaria co-infection population studies have been conducted on nine malaria-endemic countries, with heterogeneous results depending on the study design and the population or subpopulation studied (Supplementary Table S1). Most studies have evaluated malaria co-infection prevalence among COVID-19-confirmed patients, reporting a wide range of prevalence from 0.63% to 100% [18,19,20,21,22,23,25]. Among the few studies that have analyzed a whole population sample like us, the low co-infection prevalence that we found in Equatorial Guinea (0.37%) is in a similar range to what has been reported for Nigeria (0.32%) [16], India (0.73%) [27], Burkina Faso (1.9%) [15], Angola (1.9%) [17], and Gabon (2.2%) [26].

Interestingly, geographical origin was significant for both SARS-CoV-2, which was more probable in Bioko Island (the site of the country capital), and malaria, which was more probable in the Littoral Province, in Bata (a rural area) (Supplementary Table S6). Therefore, the low co-infection frequency could be due to the different distribution of the diseases, with COVID-19 being more associated with urban areas and malaria being more common in rural areas.

However, a limitation in the detection of co-infections in our study could be the small sample size obtained for the age groups of adults > 40 years (16.3%, 253/1556) and children of 5–12 years (16.8%, 261/1556), the groups most affected by COVID-19 and malaria, respectively. Indeed, in our previous study carried out in Burkina Faso, most of the co-infections were detected in children and teenagers (15), while in a study of Uganda, the highest prevalence of co-infection was in the age groups of 0–20 years and above 60 years (20). Another potential limitation of the study could be the bias during data collection due to the non-random and non-stratified nature of the sampling; that is, study participants corresponded to people attending health centres. To correct for this bias, the prevalence estimates were adjusted considering national population data for gender and age.

The low SARS-CoV-2 prevalence reported in Equatorial Guinea and the low case severity could support the hypothesis of protection by cross-immunity or trained immunity due to co-occurring endemic diseases such as malaria [46,47,48]. In this study, however, we cannot conclude the consequences of malaria and COVID-19 co-infection since the clinical course for co-infection cases was not systematically recorded. Other studies have addressed the clinical consequences of malaria and COVID-19 co-infection but with contradictory results. Retrospective studies with COVID-19 patients have found that malaria co-infection is associated with prolonged hospitalization [18] and has a greater mortality risk compared with just SARS-CoV-2 infection [25]. However, other studies report that patients with SARS-CoV-2 and malaria do not seem to have a worse disease outcome, but previous malaria exposure appears to be related to a lower frequency of severe COVID-19 [22,27]. All this considered, additional retrospective and prospective studies are required to better understand the clinical implications of SARS-CoV-2 and malaria co-infections.

## 4. Conclusions

The present study expands on the limited knowledge about COVID-19 prevalence in Central Africa. Our results provide further insights into SARS-CoV-2 variant circulation, transmission and evolution in Africa and confirm that by June to October 2021, the Delta variant AY.43 was dominating SARS-CoV-2 infections in Equatorial Guinea, together with a few minor variants. This study also challenges the assumption of Africa as a source of new variants that subsequently spread globally. In our case, the Delta variant, with its origin in India, dominates the Equatorial Guinea dataset with subsequent spread to other continents. Finally, we report SARS-CoV-2 and malaria co-infection cases at a low frequency in the population. Importantly, co-infection cases did not worsen symptoms or severity. Our study is relevant in the context of successful pandemic management and preparedness, which need to be based on quality epidemiological data from previous pandemics and proper national genomic surveillance programmes.

## Figures and Tables

**Table 1:** Distribution and demographic characteristics of positive cases in the whole sampled population by age and gender.

Age Group (Years)	Gender ^a^	Non-Infected	SARS-CoV-2	Malaria	SARS-CoV-2 and Malaria	Subtotal	Total
A. 5–12	F	89 (62.67%)	4 (2.81%)	49 (34.51%)	0	142 (54.41%)	
	M	69 (57.98%)	5 (4.20%)	47 (39.49%)	2 (1.68%)	119 (45.59%)	261 (16.77%)
B. 13–20	F	149 (61.08%)	8 (3.33%)	84 (35.00%)	1 (0.42%)	240 (74.30%)	
	M	54 (65.06%)	1 (1.22%)	28 (33.73%)	0	83 (25.69%)	323 (20.76%)
C. 21–40	F	444 (79.14%)	11 (1.96%)	108 (19.25%)	2 (0.36%)	561 (78.02%)	
	M	128 (81.01%)	9 (5.69%)	21(13.29%)	0	158 (21.97%)	719 (46.21%)
D. >40	F	152 (81.72%)	5 (2.69%)	30 (16.13%)	1 (0.54%)	186 (73.52%)	
	M	52 (77.61%)	3 (4.48%)	12 (17.91%)	0	67 (26.48%)	253 (16.26%)
Subtotal	F	834 (73.87%)	28 (2.48%)	271 (24.00%)	4 (0.35%)	1.129 (72.56%)	
	M	303 (70.96%)	18 (4.21%)	108 (25.29%)	2 (0.47%)	427 (27.44%)	
Total		1.137 (73.07%)	46 (2.96%)	379 (24.36%)	6 (0.39%)		1.556

Data are presented as n (crude prevalence %). ^a^ F, female; M, male.

## Data Availability

The datasets generated for this study can be found in the European Nucleotide Archive (ENA) database with project ID PRJEB65711 and sample ID ERS16307344-ERS16307386.

## Supplementary Materials

The following supporting information can be downloaded at: https://www.mdpi.com/article/10.3390/idr17050111/s1, Table S1. Representative SARS-CoV-2 and malaria co-infection published population studies; Table S2. Distribution of samples by sampling locality; Table S3. Prevalence of infections adjusted by age and gender considering national data from 2021; Table S4. Presence of signs and symptoms in the whole sampled population; Table S5. Description of signs and symptoms in the SARS-CoV-2, malaria, and co-infected symptomatic sub-population; Table S6. Estimates of multivariable binomial regression model; Table S7. Circulating lineages of SARS-CoV-2 in Equatorial Guinea from June to October 2021.
