# Supplementary material for: Circulation of SARS-CoV-2 and Co-Infection with Plasmodium falciparum in Equatorial Guinea
Source: Infect Dis Rep. 2025 Sep 10;17(5):111. doi: 10.3390/idr17050111 (PMC12452662; doi:10.3390/idr17050111)
Supplement: Supplementary file 1 [file idr-17-00111-s001.zip › idr-3713897-supplementary.pdf]

Circulation of SARS-CoV-2 and co-infection with Plasmodium falciparum in Equatorial Guinea

Supplementary Materials

Table S1. Representative SARS-CoV-2 and malaria co-infection published population studies.

Reference	Study location (sampling dates)	Study design	Participants (N)	COVID-19 (n)	Malaria coinfection (n)	Malaria prevalence among COVID-19	Overall co-infection prevalence	Clinical data
Amoo 2020	Lagos, Nigeria, (April-May 2020)	Cross-sectional study.	Patients suspected with COVID-19 (617)	121	2	1.66%, 2/121	0.32%, 2/617	Asymptomatic (52%), mild (48%)
Muhammad 2020	Dutse, Nigeria (March-July 2020)	Cross-sectional study	Participants (74): patients with COVID-19 (54), healthy controls (20)	54	34	62.9%, 34/54	45.9%, 34/74	NS
Onosakponome 2020	Rivers State, Nigeria (2020)	Cross-sectional study	Patients with COVID-19 (300)	300	300	100%, 300/300	ND	NS
Matanguila 2020	Kinshasa, Democratic Republic of Congo	Retrospective cohort study	Patients with COVID-19 (160)	160	1	0.63%, 1/160	ND	Mild (57%), moderate (12%), severe (31%)
Achan 2022	Uganda (April-October 2020)	Exploratory prospective	Patients with COVID-19 (597)	597	70	12%, 70/597	ND	Asymptomatic (43%), mild (39%), moderate (8%), severe (8%), critical (3%)
Bakamutumako 2021	Uganda (March – December 2020)	Prospective cohort Study	Patients with COVID-19 (270)	270	4	1.5%, 4/270	ND	Symptomatic, mildly symptomatic, asymptomatic
Morton 2021	Malawi (April-September 2020)	Prospective cohort Study	Patients suspected with COVID-19 (87)	66 (41 PCR+, 25 IgG+/PCR-)	3	4.5%, 3/66	3.45%, 3/87	Severe acute respiratory infection (SARI)
Mahajan 2021	Mumbai, India (April-October 2020)	Retrospective cohort study	Front-line health-care workers (3.711)	491	27	5.5%, 27/491	0.73%, 27/3.711	Mild (73.9%), moderate (12.5%), severe (2.4%)
Hussein R. 2022	Khartoum, Sudan (May-December 2020)	Retrospective cohort study	Patients with COVID-19 (591)	591	270	45.7%, 270/591	ND	Symptomatic.
Ditombi 2022	Gabon (October-	Cross-sectional study	Febrile patients (children and adolescents) (135)	10	3	30%, 3/10	2.2%, 3/135	Symptomatic.

	December 2021)							
Sebastião 2022	Luanda, Angola (April 2021)	Cross-sectional study	105 subjects	4	2	50%, 2/4	1.9% 2/105	NS
López-Farfán 2022	Burkina Faso (August-November 2020)	Cross-sectional study	Random asymptomatic volunteers (998)	49	19	38,8%, 19/49	1.9%, 19/998	Asymptomatic
Ingabire 2022	Uganda (January-December 2021)	Retrospective cohort study	Patients with COVID-19 (968)	968	70	7.2% (70/968)	ND	Mild (7%), moderate (43%), severe (46%), critical (4.1%)

NS: not specified

Table S2. Distribution of patients by sampling locality.

Locality	Health Centre	Samples (n)	Malaria (n)	SARS-CoV-2 (n)	Co-infection (n)
Bata	Hospital Regional de Bata	309	45	1	1
	Centro de Salud María Rafols	489	211	1	1
	Centro de Salud La Libertad	115	24	1	1
Bioko Island	Centro de Salud Buena Esperanza	412	84	27	3
	Centro de Salud de Campo Yaundé	203	15	16	0
	Hospital Regional de Malabo	28	0	0	0
Total		1.556	379	46	6

Table S3. Prevalence of infections adjusted by age and gender, considering national data from 2021 (35)

	Prevalence (%)	Adjusted Prevalence (%)	95% CI	SE
Malaria	24.36	22.25	20.09 – 24.41	1.1
SARS-CoV-2	2.96	3.03	2.11 – 3.94	0.47
Co-infection	0.39	0.37	0.01 – 0.69	0.02

Table S4. Presence of signs and symptoms in the whole sampled population.

	Non-infected* (n=1.137)	SARS-CoV-2 (n=46)	malaria (n=379)	Co-infected (n=6)
**Symptomatic**	758 (66.67%)	42 (91.30%)	350 (92.35%)	5 (83.33%)
**Asymptomatic**	342 (30.08%)	3 (6.52%)	13 (3.43%)	0
**NA****	37 (3.25%)	1 (2.17%)	16 (4.22%)	1 (16.67%)
**Pregnant**	78 (6.86%)	2 (4.34%)	17 (4.48%)	1 (16.67%)

Data are presented as n (%).
*non-infected: persons negative for malaria and SARS-CoV-2.
**NA: not annotated.

Table S5. Description of signs and symptoms in the SARS-CoV-2, malaria and co-infected symptomatic sub-population.

Signs and symptoms	SARS-CoV-2 (n=42)	Malaria (n=350)	Co-infection (n=5)
Not specified	25 (59.52%)	52 (14.85%)	2 (40,00%)
Fever	16 (38.09%)	153 (43.71%)	2 (40,00%)
Fever and others*		131 (37.43%)	
Weakness, headache, dizziness and muscle pains	1 (2.38%)	9 (2.57%)	1 (20,00%)
Vomiting and others (weakness, headache)		5 (1.43%)	

Data are presented as n (%).
*Headache, weakness, muscle and joint pain, cough, dizziness, vomiting, diarrhoea, anorexia.

Table S6. Estimates of Multivariable binomial regression model. Samples were excluded because they missed temperature information, so sample size was 1476.

	Estimate	P – value
**Model – malaria**		
Age	-0.028	< 0.001
Sex – Male	0.031	0.839
COVID infection	-0.931	0.092
Fever > 37ºC	2.165	< 0.001
Origin – Litoral Province	0.874	< 0.001
**Model – SARS-CoV-2**		
Age	0.006	0.553
Sex – Male	0.446	0.177
Malaria infection	-0.826	0.134
Fever > 37ºC	0.829	0.021
Origin – Littoral Province	-4.024	< 0.001
**Model – Coinfection**		
Age	-0.029	0.494
Sex – Male	0.673	0.508
Fever > 37ºC	17.036	0.993
Origin – Littoral Province	-1.471	0.205

Table S7. Circulating lineages of SARS-CoV-2 in Equatorial Guinea from June to October 2021.

Lineage	Samples (n)	Malaria co-infection (n)
AY.43	38	4
AY.36	3	0
B.1.351	2	1
